# Exploring Determinants of Spatial Variations in the Dengue Fever Epidemic Using Geographically Weighted Regression Model: A Case Study in the Joint Guangzhou-Foshan Area, China, 2014

**DOI:** 10.3390/ijerph14121518

**Published:** 2017-12-06

**Authors:** Hongyan Ren, Lan Zheng, Qiaoxuan Li, Wu Yuan, Liang Lu

**Affiliations:** 1 State Key Laboratory of Resources and Environmental Information System, Institute of Geographic Sciences and Natural Resources Research, Chinese Academy of Sciences, Beijing 100101, China; zhenglan1007@163.com (L.Z.); liqx@lreis.ac.cn (Q.L.); 2 Key Laboratory of Geographic Information Science, Ministry of Education, East China Normal University, Shanghai 200241, China; 3 College of Geographical Science, Fujian Normal University, Fuzhou 350007, China; 4 School of Computer Science and Technology, Beijing Institute of Technology, Beijing 100081, China; yuanwu@bit.edu.cn; 5 Department of Vector Biology and Control, Natural Institute for Communicable Disease Control and Prevention, Chinese Center for Disease Control and Prevention, Beijing 102206, China

**Keywords:** dengue fever, spatial variations, Guangzhou and Foshan, socioeconomic factors, geographically weighted regression

## Abstract

Dengue fever (DF) is a common and rapidly spreading vector-borne viral disease in tropical and subtropical regions. In recent years, this imported disease has posed an increasing threat to public health in China, especially in many southern cities. Although the severity of DF outbreaks in these cities is generally associated with known risk factors at various administrative levels, spatial heterogeneities of these associations remain little understood on a finer scale. In this study, the neighboring Guangzhou and Foshan (GF) cities were considered as a joint area for characterizing the spatial variations in the 2014 DF epidemic at various grid levels from 1 × 1 km^2^ to 6 × 6 km^2^. On an appropriate scale, geographically weighted regression (GWR) models were employed to interpret the influences of socioeconomic and environmental factors on this epidemic across the GF area. DF transmissions in Guangzhou and Foshan cities presented synchronous temporal changes and spatial expansions during the main epidemic months. Across the GF area, this epidemic was obviously spatially featured at various grid levels, especially on the 2 × 2 km^2^ scale. Its spatial variations were relatively sufficiently explained by population size, road density, and economic status integrated in the GWR model with the lowest Akaike Information Criterion (AICc = 5227.97) and highest adjusted R square (0.732) values. These results indicated that these three socioeconomic factors acted as geographical determinants of spatial variability of the 2014 DF epidemic across the joint GF area, although some other potential factors should be added to improve the explaining the spatial variations in the central zones. This work improves our understanding of the effects of socioeconomic conditions on the spatial variations in this epidemic and helps local hygienic authorities to make targeted joint interventions for preventing and controlling this epidemic across the GF area.

## 1. Introduction

Dengue is a vector-borne tropical disease caused by the four dengue virus serotypes (DENV 1–4) that is mainly transmitted by *Aedes aegypti* and *Aedes albopicuts* mosquitoes. Dengue is an endemic infectious disease in most of the tropical and subtropical regions, especially in Southeast Asia, the Western Pacific, Latin America, and Eastern Mediterranean regions [1]. With increased geographical extension, number of cases, and disease severity, dengue has become a worldwide public health issue due to the unprecedented growth and migration of the population, excessive urbanization, and the spread of mosquito vectors [2,3]. 

There was no dengue fever (DF) case documented in China from 1949 to 1977 until an outbreak emerged in Guangdong Province in 1978. DF is still believed to be an imported infection in China, although the threat of DF outbreaks has become more and more serious over the past 15 years, especially in areas of Southern China including Guangdong, Zhejiang, Yunan, Fujian, and Guangxi Provinces [4,5,6,7]. In these non-endemic regions, the imported cases from the infected areas commonly trigger and even determine the size of the DF outbreaks, especially when coupled with abnormal meteorological conditions (e.g., extreme rainfall) [8,9,10]. 

Once the DF outbreaks occur in the non-endemic regions, local DF transmission likely depends on the interactions between host, viruses, mosquitoes, and environmental factors [1,5]. Given that the range of a mosquito’s flying distance is restricted (commonly 512 m or less [11,12]), local DF transmission is commonly affected by the spatially differentiated density and movement of the local population on broad (national or international) or finer (regional, county, community, or neighborhood) spatial scales [11,13,14,15]. In highly urbanized regions, the population tends to be more crowded and more likely to be bitten by *Aedes albopictus* mosquitoes (dominant species in the Pearl River Delta (PRD)) due to the suitable settings [16,17,18,19]. In addition to an effective early warning system, which is urgently demanded to predict the possible DF outbreaks in the non-endemic areas [20], revealing the influencing factors of spatial patterns of the DF incidence rates is seemingly more important for sufficiently making and implementing targeted prevention and control measures. 

As one of the most economically developed regions, the Pearl River Delta (PRD) is frequently confronted with a serious situation of DF prevention and control because it has the most cases—accounting for more than 90%—in China [21,22]. DF epidemics in the Guangzhou City and Foshan City in the PRD core area have commonly been affected by each other [5,23,24] due to the increasingly indistinguishable boundary between these two cities caused by parallel socioeconomic development and the closer and closer relationship of citizens’ living, working, and communicating during the “*Guangzhou and Foshan (GF) to be One*” project.

Previous studies that focused on the causes and influencing factors of the DF epidemic have improved our understanding of the causes of the increasingly serious infection situation in the south of China [5,10,22]. However, spatial patterns of DF epidemics and their determinants remain little understood for these two cities as an integral (joint GF) area. Therefore, this study aimed to (1) characterize spatial patterns of the 2014 local DF epidemic on an appropriate scale in this joint area, and to (2) explore their responses to spatially homogeneous socioeconomic factors using the geographically weighted regression (GWR) model. This study provides helpful clues for local public health authorities in establishing effective measures for DF prevention and control.

## 2. Methods and Materials

### 2.1. Study Area

**Figure 1:**
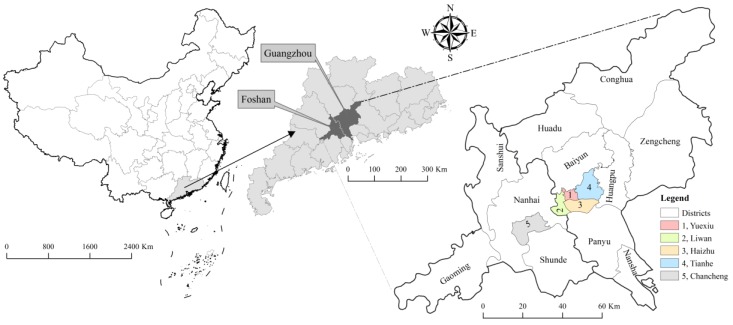
Illustration of the joint Guangzhao–Foshan (GF) area with five core urban districts highlighted by different colors.

Guangzhou City (112°57′~114°0′ E, 22°34′~23°57′ N) and Foshan City (112°23′~113°23′ E, 22°38′~23°35′ N) have become more closely bound as a core zone and arelabeled the joint GF area in the central region of Guangdong Province (Figure 1) due to the “*Guangzhou and Foshan to be One*” project. The area, which has a population of about 21 million and had the economic level of gross domestic product (GDP) per capita of about 125,000 yuan in 2015, is featured by warm, humid, and rainy meteorological conditions of the maritime monsoon climate.

Compared with the county, town, village, or district scale used as basic geographic units in previous spatial epidemiology studies, the instability of calculating or processing research objects is subject to disturbances caused by the irregularity and nonconformity of constantly changing administrative divisions [4,5,22,25]. This phenomenon can be efficiently avoided by using regular spatial grid analysis [26]. In this study, a series of spatial grids including 1 × 1 km^2^, 2 × 2 km^2^, 3 × 3 km^2^, 4 × 4 km^2^, 5 × 5 km^2^, and 6 × 6 km^2^ were created with the Fishnet Tool in ArcGIS 10.0 (ESRI, Redlands, CA, USA).

### 2.2. Data Collection

#### 2.2.1. DF Incidence Data 

**Figure 2:**
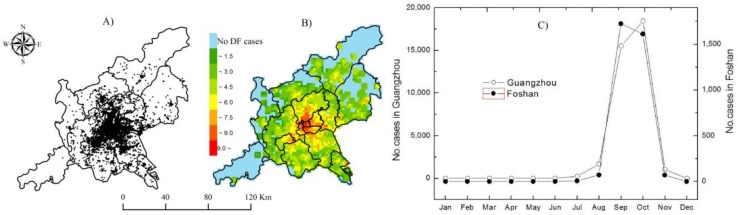
Spatial distribution and temporal variations in the 2014 dengue fever (DF) epidemic (**A**): reported cases; (**B**): Natural logarithm of the spatially empirical Bayes (SEB)-smoothed DF incidence rates; （**C**）: monthly changes).

The 2014 epidemic data of confirmed dengue cases, such as hospital information, patient addresses, and recent trip records on overseas and domestic travel, in the joint GF area was obtained from the China Notifiable Disease Surveillance System. Using a geocoding technique (http://www.gpsspg.com/xGeocoding/), all of the local cases without recent travel experience were spatially positioned according to their addresses (Figure 2A). The 2014 dengue cases at various grid scales were counted based on the above-mentioned data. Then, the ratio of the DF cases to the population (noted as the incidence rates of DF at these gridded scales) was calculated and smoothed by the spatially empirical Bayes (SEB) method in GeoDa (OpenGeoDa 1.2.0, Spatial Analysis Laboratory, Urbana, IL, USA, 2012) to decrease the spatial instability of the gridded DF incidence rates. Natural logarithm values of the SEB-smoothed DF incidence rates were also calculated for further spatial analysis and modeling as shown in Figure 2B. 

#### 2.2.2. Socioeconomic and Environmental Data

Since the specific impacts on the host and vector mosquitoes during the DF transmission [27], four socioeconomic variables (land urbanization level (LUL), population size, road density, and gross domestic product (GDP)) and four environmental factors (normalized difference of vegetation index (NDVI), mean temperature (MT), mean precipitation (MP), and mean relative humidity (MRH)) were obtained from some open nonprofit data sources and the data treatments were described in detail in Table 1. 

### 2.3. Spatial Autocorrelation Analysis

As a typically useful tool, spatial autocorrelation analyses are frequently utilized to explore the spatial patterns of incidence or mortality in terms of Moran’s I with z-score and/or *p*-value because of its greater statistical power [28,29,30]. Moran’s I is produced by standardizing the spatial autocovariance by the variance of the data using a measure of the connectivity of the data [31]. Generally, Moran’s I values range from −1 to 1 and a high positive Moran’s I value with larger z-score and/or appropriate *p*-value represents a tendency towards clustering, which means that adjacent grids have similar levels of incidence rate, whereas a low negative value indicates a tendency towards dispersal, which means that grids with high incidence rate lie next to grids with low incidence rate. A more complete description is provided by Anselin and Getis [31].

### 2.4. Geographically Weighted Regression Modeling

Given the spatiotemporal heterogeneity of dengue fever incidence rates, the related factors may affect the epidemic in different ways and to various degrees, which is appropriate to analyze using a geographically weighted regression (GWR) model. As an extension of the traditional multiple linear regression (i.e., ordinary least square, OLS), a GWR model embeds the attributes’ spatial location into the regression parameter, yielding a local regression together with local estimates of regression coefficients [32]. The local estimation of the parameters with GWR is expressed by Equation (1) as below:

(1) $$y_{i}=\beta_{0}\left(u_{i},v_{i}\right)+\sum_{k=1}^{n}\beta_{k}\left(u_{i},v_{i}\right)x_{ik}+\varepsilon_{i}\;(i=1,2,\ldots ,\;m)$$

where *i* = 1, 2,…, m denotes the number of spatial grids in the joint GF area; $y_{i}$ is the dependent variable (dengue fever incidence rate) at location *i*; independent variable $x_{ik}$ is the value of the *k* parameter at location *i*, $x_{ik}$ referred to the value of an affecting factor *k* (such as LUL, economic condition, meteorological factors, and so on) at spatial grid *i*, which is specific for every spatial grid; $\beta_{0}$ is the intercept; $\beta_{ik}$ is the correlation coefficient for the independent predictor variable $x_{ik}$, which is to be estimated; and $\varepsilon_{i}$ represents random error. Then, every spatial grid has a set of specific parameters to reflect the relationship between dengue fever incidence rate and influencing factors. Finally, all the parameters derived from both GWR and OLS will be compared in terms of the corrected Akaike Information Criterion (AICc) and adjusted R square, on which the performance of these two models could be evaluated. 

In this study, Pearson correlation analysis was chosen to explore the relationships between DF incidences and all of the potential variables (climatic, socioeconomic, and vegetation conditions) at the significance level of 0.05 and 0.01, by which some appropriate potential variables could be accordingly considered into the GWR and/or OLS models.

All of the above spatial analysis and modeling were completed in ArcGIS 10.0 software (ESRI, Redlands, CA, USA). Typical correlation analysis was achieved using SPSS 14.0 (SPSS Inc., Chicago, IL, USA).

## 3. Results

### 3.1. Temporal and Spatial Distribution of Local DF Epidemic

**Figure 3:**
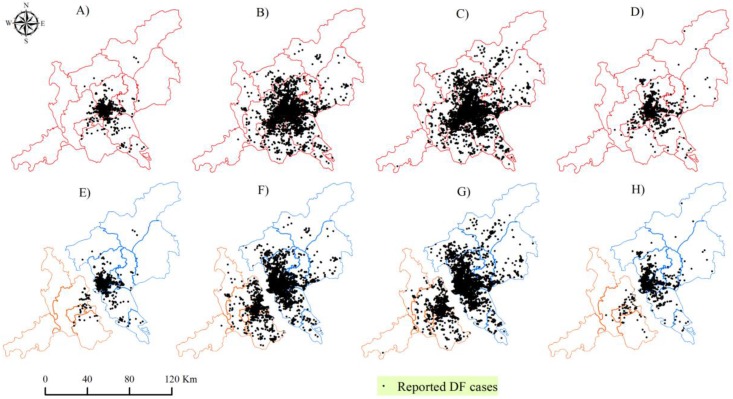
Comparison of monthly reported DF cases in the joint GF area (**A**–**D**) and Guangzhou City, Foshan City (**E**–**H**) from August to November.

In total, there were 40,450 locally infected patients confirmed by laboratory diagnosis in Guangzhou City and Foshan City. Their local DF epidemic presented similar temporal changes (Figure 2C). The overwhelming majority of local DF cases in these two cities appeared in the main epidemic months with a typical increasing in August, an obvious peaking in September–October, and a sudden decreasing toward November 2014. Moreover, the distribution of local DF cases displayed clear spatial expansion during these main epidemic months and contraction (Figure 3A–D). When the DF epidemic in each city was individually focused, these synchronous features of this epidemic in these two cities would not be exactly achieved due to the artificially isolated distributions of DF epidemics caused by administrative boundary (Figure 3E–H). This means that taking these two cities as a joint area was conducive to characterizing the real temporal and spatial patterns of the DF epidemics in the core DF epidemic area across China. 

At the spatial levels from 1 km × 1 km to 6 km × 6 km, the natural logarithm values of SEB-smoothed incidence rates presented obvious spatial clustering across the GF area, especially on the 2 km × 2 km scale, due to relatively high Morans’ I and larger z-score values (Table 2). On this scale, the spatial distribution of the 2014 local DF epidemic in these two cities was further clearly characterized through spatial analysis. The grids with higher natural logarithm values of the SEB-smoothed DF incidence rates were mainly distributed in the central and boundary districts, like Chancheng and Nanhai in Foshan City, Yuexiu, Haizhu, Tianhe, Liwan, Baiyun, and Panyu in Guangzhou City (Figure 2B). 

### 3.2. Spatial Variability of Socioeconomic and Environmental Factors

**Figure 4:**
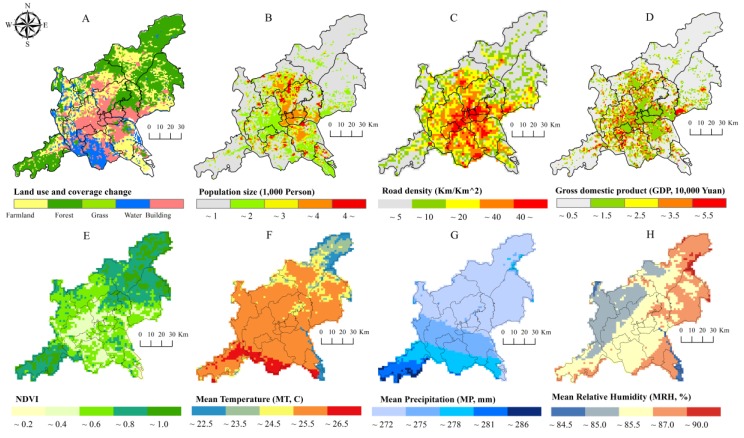
Spatial distribution of socioeconomic variables (**A**）: LUL; （**B**）: population size; （**C**）: road density; （**D**）: economic condition) and environmental factors (**E**）: NDVI; （**F**）: mean temperature; （**G**）: mean precipitation; (**H**): mean relative humidity) across the GF area.

In comparison, spatial differentiations of socioeconomic variables (Figure 4A–D) were more obvious than those of environmental factors (Figure 4E–H) across the GF area. The coefficients of variation (CV) of the socioeconomic variables were larger than those of environmental factors (Table 3). Among the four socioeconomic and four environmental variables, LUL and NDVI respectively possessed the highest CV. In addition, the natural logarithm values of SEB-smoothed DF incidence rates tended to be more closely associated with the socioeconomic variables (Table 3). These results displayed that the socioeconomic conditions and vegetation index could be preferentially considered as potential explanatory variables of the 2014 DF epidemic across the GF area.

### 3.3. Spatial Modeling 

Based on the selected explanatory variables (NDVI, LUL, GDP, population size, and road density) at the 2 km × 2 km level, the GWR models accounted for larger proportion of the spatial variations in the 2014 DF incidence rates than that of OLS models in terms of the values of higher adjusted R square and lower AICc (Table 4). 

The interpreting abilities of GWR models tended to be different from each other due to various combinations of these selected variables. When discarding NDVI, the GWR models (B–F) possessed better capacity (0.623 < Adjusted R square < 0.732) of simulating the spatial variations. While GDP, population size, or road density was further deleted respectively, the performances of the GWR models (D–F) were obviously decreased. In comparison, the GWR model C displayed the best performance (Adjusted R square = 0.732) of interpreting the spatial variations while only LUL was further discarded. In addition to the highest adjusted R square and the lowest AICc values, the range of local R square values derived from the GWR model C was the widest as shown in Table 4. 

**Figure 5:**
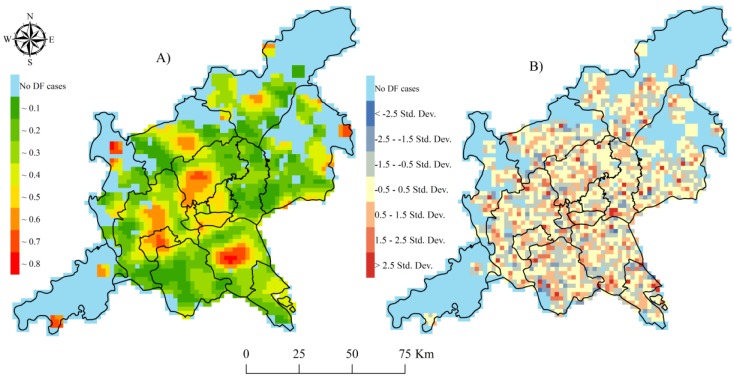
Local R square values (**A**) and standard residual values (**B**) derived from multivariate GWR model C integrating GDP, population size, and road density.

The 2014 DF incidence rates were spatially interpreted in the districts such as Nanhai and Chancheng in Foshan City and Baiyun, Liwan, Tianhe, and Haizhu in Guangzhou City due to the clustering grids with relatively higher local R square than 0.4 (Figure 5A). The grids with the standardized residual (StdResid) values in the range of −2 to 2 accounted for 95.10% of the GF area although some unusually high or low StdResid values were still observed (Figure 5B). The results implied that a stable and reliable relationship was achieved in the GWR model C by integrating GDP, Population size, and road density for the explanation of spatial variations in the 2014 DF epidemic across the GF area.

**Figure 6:**
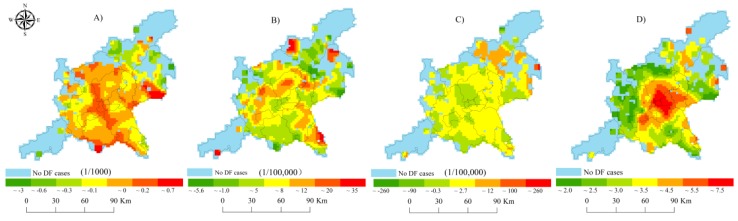
Local coefficients for population size (**A**), road density (**B**), GDP (**C**), and the intercept (**D**) derived from the GWR model C.

In addition, the strength of the local correlation between population size, road density, economic status, and this epidemic was spatially differentiated across the GF area (Figure 6). Population size possessed strongly positive effects in this epidemic, especially on the boundary zones between Guangzhou City and Foshan City (Figure 6A). Similarly, road density had strongly positive impacts on this epidemic, which resulted in an inverse U-shaped belt covering the grids with higher coefficients (Figure 6B). By contrast, the effects of the economic level (GDP) on this epidemic tended to be protective in some central zones and be threatening in most of the rest zones across the GF area, although both of them were relatively feebler (Figure 6C).

## 4. Discussion

In this study, we analyzed the influences of socioeconomic and environmental factors on the spatial variations on the grid scale in the 2014 DF epidemic in the joint GF (both Guangzhou City and Foshan City) for the first time in China. GWR models were employed to effectively explore the determinants of spatial variability of this epidemic. Several notable findings were achieved and could provide meaningful clues for public health authorities implementing effective interventions on this infection. 

In our study, the monthly DF epidemics in Guangzhou City and Foshan City presented similar features of temporal changes and spatial expansion. One explanation for this phenomenon is that neighboring Guangzhou City and Foshan City possessed similar meteorological conditions, approximate population density, large commuting population (working in one city and residing in the other one) by increasingly maturing integrative traffic system, common floricultural habits, and so on. These common crucial factors determined that these two cities were confronted with a DF outbreak risk at similar levels and their epidemics tended to be affected by each other [5,23,24]. Accordingly, considering these two cities as a joint area is an effective choice to understand this epidemic and to establish a joint intervention system in this region. Furthermore, our study showed that the 2 km × 2 km grid was the most suitable scale for characterizing the spatial variability of the 2014 DF epidemic across the GF area. We cautiously and optimistically speculate that the joint DF intervention system could be effectively implemented on this scale for preventing and controlling this disease across the GF area, especially during the continuously promoted “*Guangzhou and Foshan to be One*” project. 

The outbreak size and peaks of the 2014 DF epidemic in Guangzhou City were mainly determined by the increasing imported DF cases and much heavier rainfall in May and August [10]. After the main inducements of this epidemic were clearly revealed, the next important concern is to explore the determinants of the spatial variations in this epidemic in a special region, so as to make effective interventions for preventing and controlling this disease. Using generalized additive model, Qi, et al. [22] found that the 2013 DF epidemic (a small-scale) across the PRD area in Southern China was spatially affected by road density, population density, and GDP per capita at the township level. It was similar to our finding, but the results derived from the GWR models applied in this study was more reasonable since the spatial correlation between this epidemic and the influencing factors had been considered at a fine spatial scale. It was interesting that LUL was not included in the best-performing GWR model C but the second-performing GWR model B, which may be related to the collinearity between it and the other socioeconomic variables (Table S1). Nevertheless, these results indicated that the spatial variations in this epidemic across the GF area were heavily determined by socioeconomic factors, especially population size, road density, and economic level. 

**Figure 7:**
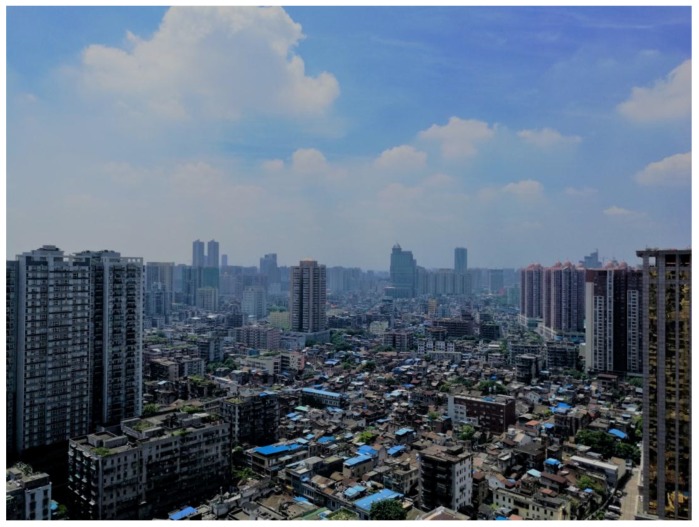
Typical urban villages with numerous crowded and low-rise developments surrounded by high buildings.

Besides the most serious DF epidemic being across the GF area, the central zones were also obviously featured by higher road density, moderate economic status, and a more crowded population (Figure 4). According to the results derived from the GWR model C, the spatially differentiated population size acted as a powerful risk factor for the 2014 DF epidemic, especially in the boundary zones between Guangzhou and Foshan City (Figure 6A), which may be related to the increasing probability of being bitten by *Aedes albopictus* mosquitoes due to crowded population [11,13,15,33]. Road density also had a risky impact on this epidemic along the inverse-U shaped belt around the central zones (Figure 6B), which was probably associated with the better traffic accessibility (high road density) for people commuting to and from affected areas and being infected by this disease [13,24]. Together with larger population size, higher road density resulted in extensive impervious surfaces in the central zones, which would easily form widely distributed plashes and supply suitable settings for *Aedes albopictus* mosquitoes’ breeding, growth, and perching, especially after a moderate rainfall [16,17,18,19,34]. In comparison, the protective and/or threatening effects of economic status on this epidemic were relatively feeble across the GF area in this study (Figure 6C), which is somewhat different from previous studies wherein high economic status means better health conditions [22,35,36]. A reasonable explanation is that the widely distributed urban villages (Figure 7) in the central districts were probably suitable for this epidemic due to a large floating population, dense low buildings, and poor sanitation [37,38,39,40]. Although these explanatory variables made important contributions for interpreting the spatial clustering DF epidemic, there were some variations in this epidemic to be explained due to its relatively high intercept values in the central zones (Figure 6D). Nevertheless, these results implied that the DF infection was more possibly prevalent in the central zones of the joint GF area. In other words, the central zones tended to be heavily confronted with higher risk of this epidemic. We accordingly suggest that some stronger and targeted interventions should be implemented in these regions by local hygienic and environmental departments for preventing and controlling this epidemic.

A few limitations of this study warrant mentioning. First, the suitability of the 2 km × 2 km scale for characterizing the DF epidemic during all of the other years should be further assessed, although it was the most appropriate for the most serious 2014 DF epidemic across the GF area. Second, remote sensing images should be adequately used to retrieve more information, such as more detailed and real ground temperature and humidity values and more widely distributed urban villages, by which the zones with relatively higher (>2) or lower (<−2) StdResid (Figure 5B) and high intercept (Figure 6D) values may be further and reasonably decreased; then the spatial variability of this epidemic in the joint GF area would be more sufficiently explained. Finally, more changeable or artificially controlled factors, such as vector density, effective local interventions, temporal interval during the onset to the diagnosis of each infected patient, and so on should also be included in the models to more closely approach the substances of the epidemic of this disease and make more targeted interventions in this non-endemic area. 

## 5. Conclusions

In summary, our study proved that spatially differentiated road density, population size, and economic level were the main determinants of spatial variability of DF epidemic on the grid scale in the joint GF area (Guangzhou and Foshan). This work improves our understanding of the effects of socioeconomic conditions on the spatial variations in this epidemic and helps local hygienic and environmental authorities to make targeted joint interventions for preventing and controlling this epidemic across the GF area. 

## Figures and Tables

**Table 1:** Data sources and processing of socioeconomic and environmental factors in this study.

Variables/Description	Data Processing	The Source of Data
Population size	Summing the population (persons) for each grid based on the 2010 population density data	Data Center of Resources and Environmental Science, Chinese Academy of Sciences (RESDC, www.resdc.cn)
Land urbanization level (LUL)	Calculating the area ratio of urbanized land to the grid based on the 2010 data of land use and coverage change	
Economic conditions	Summing the gross domestic product (GDP) values (RMB) for each grid based on the 2010 GDP data	
Road density	Calculating the ratio of total length of relatively low-level (town and/or district) roads to the grid (km·per·km^2^) based on the road network data	
☯ Annual mean temperature (AMT)	Annual mean values of temperature (June–November in 2014) in each grid	China Meteorological Data Service Center (CMDC, http://data.cma.cn/)
☯ Annual mean precipitation (AMP)	Annual mean values of precipitation (June–November in 2014) in each grid	
☯ Annual mean relative humidity (AMH)	Annual mean values of relative humidity (June–November in 2014) in each grid	
Vegetation index	Annual mean values of normalized difference of vegetation index (NDVI) (June–November in 2014) in each grid	https://ladsweb.nascom.nasa.gov/data

☯ Annual mean values of climatic conditions and vegetation index in June–November were calculated in each grid because the 2014 DF cases were mainly reported in these months.

**Table 2:** Spatial autocorrelation of the natural logarithm values of SEB-smoothed monthly DF incidence rates.

Gridded Scales	1 km × 1 km	2 km × 2 km	3 km × 3 km	4 km × 4 km	5 km × 5 km	6 km × 6 km
Z scores	38.78	42.89	33.83	28.23	21.73	18.64
Morans’ I	0.40 **^‡^**	0.72 **^‡^**	0.75 **^‡^**	0.78 **^‡^**	0.68 **^‡^**	0.67 **^‡^**
*p* values	<0.01	<0.01	<0.01	<0.01	<0.01	<0.01

**^‡^** denote the spatially clustering significance at the level of 0.01.

**Table 3:** Descriptive statistics and correlation analysis results for the dependent and independent variables.

Parameters	DF Incidence Rates *	LUL	GDP	Road Density	Population Size	MRH	MT	MP	NDVI
Mean	3.46	0.20	39,980.19	14,178.97	5590.28	85.41	25.49	311.31	0.52
Standard deviation	1.72	0.32	36,549.65	13,029.02	4461.10	12.05	3.62	44.22	0.17
Coefficients of variation (CVs, %)	49.61	156.87	91.42	91.89	79.80	14.11	14.20	14.20	31.72
DF incidence rates *	/	0.56 **^‡^**	0.18 **^‡^**	0.53 **^‡^**	0.28 **^‡^**	0.04	0.04	0.04	−0.18

* denotes the natural logarithm values of SEB-smoothed DF incidence rates; **^‡^** means the significance level (0.01) in the correlation analysis. MT: mean temperature; MP: mean precipitation; MRH: mean relative humidity.

**Table 4:** Key parameters derived from the geographically weighted regression (GWR) models with single explanatory variable or their combinations.

Models	Selected Explanatory Variables					OLS		GWR		
	NDVI	LUL	GDP	Population Size	Road Density	Adj R-Squared	AICc	Adj R-Squared	AICc	Local R-Square
A	Yes	Yes	Yes	Yes	Yes	0.346	6643.83	0.505	6127.77	0.16–0.48
B	No	Yes	Yes	Yes	Yes	0.345	6644.54	0.649	5518.15	0.06–0.63
C	No	No	Yes	Yes	Yes	0.289	6798.45	0.732	5227.87	0.01–0.74
D	No	Yes	No	Yes	Yes	0.327	6695.05	0.643	5535.84	0.01–0.63
E	No	Yes	Yes	No	Yes	0.346	6642.59	0.628	5614.32	0.03–0.58
F	No	Yes	Yes	Yes	No	0.321	6712.95	0.623	5634.48	0.01–0.57

Note: NDVI, normalized differences of vegetation index; LUL, land urbanization level; GDP, gross domestic product; AICc, Akaike information criterion.

## Supplementary Materials

The following are available online at www.mdpi.com/1660-4601/14/12/1518/s1, Table S1: Correlation coefficients between LUL, economic level, road density, population size, and vegetation condition.
