# Supplementary material for: Exploring Determinants of Spatial Variations in the Dengue Fever Epidemic Using Geographically Weighted Regression Model: A Case Study in the Joint Guangzhou-Foshan Area, China, 2014
Source: Int J Environ Res Public Health. 2017 Dec 6;14(12):1518. doi: 10.3390/ijerph14121518 (PMC5750936; doi:10.3390/ijerph14121518)
Supplement: Supplementary file 1 [file ijerph-14-01518-s001.zip › ijerph-247407-sup-proof.docx]

**Supplementary Materials: Exploring Determinants of Spatial Variations in the Dengue Fever Epidemic Using Geographically Weighted Regression Model: A Case Study in the Joint Guangzhou-Foshan Area, China, 2014**

**Hongyan Ren 1,*, Lan Zheng 1,2, Qiaoxuan Li 1,3, Wu Yuan 4 and Liang Lu 5,***

**Table S1.** Correlation coefficients between LUL, economic level, road density, population size, and vegetation condition.

| **Heading Column** | **LUL** | **Economic Level** | **Road Density** | **Population Size** | **Vegetation Condition** |
| --- | --- | --- | --- | --- | --- |
| **LUL** | / | 0.49 **^‡^** | 0.81 **^‡^** | 0.52 **^‡^** | −0.29 **^‡^** |
| **Economic level** |  | / | 0.53 **^‡^** | 0.57 **^‡^** | −0.30 **^‡^** |
| **Road density** |  |  | / | 0.61 **^‡^** | −0.32 **^‡^** |
| **Population size** |  |  |  | / | −0.24 **^‡^** |
| **Vegetation condition** |  |  |  |  | / |

**^‡^** means the significance level (0.01).

© 2016 by the authors; licensee MDPI, Basel, Switzerland. This article is an open access article distributed under the terms and conditions of the Creative Commons by Attribution (CC-BY) license (http://creativecommons.org/licenses/by/4.0/).
