# Supplementary material for: Exploring Determinants of Spatial Variations in the Dengue Fever Epidemic Using Geographically Weighted Regression Model: A Case Study in the Joint Guangzhou-Foshan Area, China, 2014
Source: Int J Environ Res Public Health. 2017 Dec 6;14(12):1518. doi: 10.3390/ijerph14121518 (PMC5750936; doi:10.3390/ijerph14121518)
Supplement: Supplementary file 1 [file ijerph-14-01518-s001.zip › ijerph-247407-sup-proof.pdf]

Supplementary Materials: Exploring Determinants of Spatial Variations in the Dengue Fever Epidemic Using Geographically Weighted Regression Model: A Case Study in the Joint Guangzhou-Foshan Area, China, 2014

Hongyan Ren 1,*, Lan Zheng 1,2, Qiaoxuan Li 1,3, Wu Yuan 4 and Liang Lu 5,*

Table S1. Correlation coefficients between LUL, economic level, road density, population size, and vegetation condition.

Heading Column	LUL	Economic Level	Road Density	Population Size	Vegetation Condition
**LUL**	/	0.49 ‡	0.81 ‡	0.52 ‡	−0.29 ‡
**Economic level**		/	0.53 ‡	0.57 ‡	−0.30 ‡
**Road densiy**			/	0.61 ‡	−0.32 ‡
**Population size**				/	−0.24 ‡
**Vegetation condition**					/

‡ means the significance level (0.01).

© 2016 by the authors; licensee MDPI, Basel, Switzerland. This article is an open access article distributed under the terms and conditions of the Creative Commons by Attribution (CC-BY) license (http://creativecommons.org/licenses/by/4.0/).
